# Hemorrhagic ascites as a complication of heart failure: A case report and review of the literature

**DOI:** 10.1097/MD.0000000000030708

**Published:** 2022-09-23

**Authors:** Shahem Abbarh, Amr Farwati, Nedia Neffati, Mhd Baraa Habib

**Affiliations:** a Department of Internal Medicine, Hamad Medical Corporation, Doha, Qatar.

**Keywords:** bloody ascites, heart failure, hemorrhagic ascites

## Abstract

Although ascites is a common complication of congestive heart failure, the association between heart failure and hemorrhagic ascites is quite rare.

**Patient concerns:**

A 64-year-old woman with recurrent large bloody ascites secondary to heart failure.

**Diagnosis:**

Ascitic fluid assessment revealed red blood cells of 75,125/mm^3^ and white blood cells of 225/mm^3^. The total protein in the ascitic fluid was 28.7 g/L, with a high serum ascites albumin gradient. Peritoneal fluid examinations for bacterial culture, acid-fast bacilli (smear and culture), and malignant cell cytology were negative.

**Interventions:**

The patient was managed with therapeutic paracentesis, aggressive diuresis, and optimization of her heart failure medications.

**Outcomes:**

The patient’s symptoms improved dramatically and was discharged in a stable condition.

**Conclusion:**

Congestive heart failure should be considered as a potential cause of hemorrhagic ascites after ruling out other serious causes.

## 1. Introduction

Heart failure (HF) is a complex clinical syndrome identified by characteristic fluid overload features along with evidence of cardiac dysfunction. It can result from systolic or diastolic dysfunction. The outcome of both processes is increased left and/or right ventricular filling pressures, resulting in elevation of pulmonary and/or systemic venous pressures, which may eventually lead to organ congestion. This can be manifested as dyspnea, orthopnea, elevated jugular venous pressure, pedal edema, and ascites.^[1]^

Ascites is defined as a pathologic accumulation of fluid in the peritoneal cavity. There are numerous causes of ascites, with cirrhosis being the most common cause.^[2]^ Causes of ascites can be categorized based on the underlying pathophysiology into portal hypertension, neoplastic, inflammatory, and miscellaneous.^[3]^ The International Ascites Club classifies ascites according to severity, complication, and response to diuretic treatment. Classically, ascites can be classified into grade 1 (mild: only diagnosed on ultrasound), grade 2 (moderate: clinically sensible abdominal distension), and grade 3 (large: marked or tense abdominal distention) according to severity; into uncomplicated according to the absence of complication; and into diuretic-resistant and diuretic-intractable according to the response to diuretic treatment.^[4,5]^

Ascites is a known manifestation of HF and reflects longstanding systemic venous hypertension and passive hepatic congestion. Classically, cardiac ascites is characterized by a serous appearance grossly. Bloody or hemorrhagic ascites, described in most literature as ascitic fluid with red blood cells (RBC) count >10,000/ mm^3^,^[6,7]^ is usually seen in cirrhotic patients, malignancy-related ascites, and iatrogenic ascites.^[7]^ However, hemorrhagic cardiac ascites is a true rarity, with only one case report published in the literature in 1998.^[8]^ Here, we report a case of recurrent hemorrhagic ascites secondary to HF after excluding other causes.

## 2. Case presentation

Our patient is a 64-year-old Sudanese lady with a past medical history of hypertension, type 2 diabetes mellitus, chronic kidney disease, coronary artery disease status postpercutaneous coronary intervention with stent placement 8 years before presentation, and preserved ejection fraction heart failure with recurrent admissions due to decompensation. The patient presented to the hospital with a one-week history of abdominal distension, generalized abdominal pain, and increased bilateral lower limb swelling. One month earlier, she was admitted with the same complaints, diagnosed with decompensated heart failure secondary to medication noncompliance, and discharged on furosemide 40 mg twice daily.

On examination, the patient’s vital signs were within normal range apart from blood pressure of 150/77 mm Hg. Chest examination revealed bilateral equal air entry without crackles or wheezes. Cardiovascular examination showed biventricular S3, loud P2, and a holosystolic murmur in the left lower parasternal border. Jugular venous pressure was elevated with positive hepatojugular reflux. The abdomen was severely distended, with moderate tenderness on the right upper quadrant. Shifting dullness was positive, and no hepatosplenomegaly was detected. Severe bilateral pitting pedal edema was also appreciated.

Blood investigations (**Table 1**) revealed chronic anemia and stable renal function tests around her baseline. A 24-hour urinalysis revealed a protein of 0.57 g/24 hours. Chest X-ray showed cardiomegaly, prominent bronchovascular markings, and clear costophrenic angles. Electrocardiogram revealed left axis deviation with no significant new changes. Transthoracic echocardiography (TTE) showed left ventricular ejection fraction of 56%, moderately dilated right ventricle, and severely dilated left and right atria. TTE also showed mild mitral regurgitation, severe tricuspid regurgitation, and severely increased pulmonary artery pressure at 71 mm Hg. On abdominal ultrasound, hepatomegaly of 19.4 cm with fatty liver infiltration, and large ascites were appreciated.

**Table 1:** Blood test results.

Blood tests	Results	Reference range
HGB (gm/dL)	10	12–15
WBC (10^3^/µL)	8	4–10
Plt (10^3^/µL)	222	150–400
Cr (μmol/L)	154	44–80
BUN (mmol/L)	12.5	2.5–7.8
Na (mmol/L)	138	133–146
K (mmol/L)	4.9	3.5–5.3
NT-proBNP (pg/mL)	6434	<125
ALT (U/L)	6	0–33
AST (U/L)	15	0–32
ALP (U/L)	123	35–104
Bilirubin (μmol/L)	13	0–21
INR	1.2	1
Albumin (gm/L)	31	35–50
Total protein (gm/L)	58	60–80

ALP = alkaline phosphatase, ALT = alanine transaminase, AST = aspartate transaminase, BUN = blood urea nitrogen, Cr = creatinine, HGB = hemoglobin, INR = international normalized ratio, K = potassium, Na = sodium, NT-proBNP = N-terminal pro-brain-type natriuretic peptide, Plt = platelets, WBC = white blood cells.

Ultrasound-guided pigtail drainage catheter was inserted for abdominal paracentesis. Peritoneal fluid was bloody upon gross examination. Ascitic fluid assessment (**Table 2**) revealed RBC of 75,125/mm^3^ and white blood cells of 225/mm^3^ (neutrophils 4%, lymphocytes 49%, macrophages 43%, and monocytes 3%). The total protein in the ascitic fluid was 28.7 g/L, with an albumin of 17.2 g/L and a high serum ascites albumin gradient (SAAG) at 14.8 g/L. Bacterial culture as well as acid-fast bacilli (AFB) smear and culture from the peritoneal fluid were negative. Cytology, looking for malignant cells, was also negative. Interestingly, abdominal paracentesis was done during the previous admission and also revealed grossly hemorrhagic body fluid with RBC of 115.250/mm^3^ and similar other lab parameters. Peritoneal fluid examinations for bacterial culture, AFB (smear and culture), and malignant cell cytology were also negative at that time. A whole-body positron emission tomography (PET) scan was done to rule out secondary causes of hemorrhagic ascites, such as malignancy, and was unremarkable.

**Table 2:** Ascites fluid analysis.

Ascitic fluid analysis	First presentation (1 mo ago)	Second presentation (current admission)
RBC/mm^3^	115.250	75,125
WBC/mm^3^	81	225
Neutrophils %	8	4
Lymphocytes %	69	49
Total protein (g/L)	27	28.7
Albumin (g/L)	16.5	17
SAAG (g/L)	15	14
LDH (U/L)	106	94
Glucose (mmol/L)	11.9	9.4
Amylase (U/L)	11	7
Lipase (U/L)	15	13.9
Triglyceride (mmol/L)	0.3	0.4

LDH = lactate dehydrogenase, RBC = red blood cells, SAAG = serum ascites albumin gradient, WBC = white blood cells.

The patient was managed as a case of decompensated right-side HF and was started on intravenous furosemide and metolazone. During her hospital stay, a total of 17 liters of peritoneal fluid were drained. Subsequently, the patient’s symptoms improved dramatically, and she was discharged home on a higher dose of furosemide (80 mg thrice daily), with regular follow-ups in heart failure clinic.

## 3. Discussion

Cardiac cirrhosis is a term that includes the spectrum of hepatic disorders that occur secondary to hepatic congestion due to cardiac dysfunction, especially in the right heart chambers. It may manifest as pulsatile hepatomegaly, ascites, or cirrhosis.^[9]^ The pathogenesis behind this has been explained thoroughly in the literature. The elevated preload or central venous pressure is transmitted from the right heart chambers to the hepatic veins and sinusoids, leading to intrahepatic edema, decreased perfusion, and oxygen diffusion, as well as hemorrhagic injury and modification of the hepatocyte architecture and atrophy with associated collagen deposition, and eventually fibrosis to the hepatic veins and sinusoids.^[9]^

Clinically, ascites is attributed to heart failure in the setting of chronic cardiac disease with clinical features of overload (such as elevated jugular venous pressure, pulmonary congestion, and peripheral edema) but without predisposing factors for cirrhosis. Hepatojugular reflux is a physical sign usually present in hepatic congestion and can help differentiate cardiac ascites from other causes. Our patient is known to have a significant past cardiac history with remarkable right-side HF features that were evident on clinical examination. On the other hand, she has no apparent risk factors for cirrhosis, and no stigmata of chronic liver disease were detected on examination.

Lab-wise, cardiac ascites exhibit a characteristic pattern in its laboratory parameters, being an exudate, according to the past ascites classifications, but one with a high serum ascites albumin gradient (SAAG).^[10]^ As both cardiac and cirrhotic ascites are high-SAAG fluids, Runyon et al^[2]^ suggested using alpha-fetoprotein (AFP) of >2.5 g/dL to differentiate the 2 conditions, which has been validated by multiple studies.^[11,12]^ In the case of ascites from cirrhosis, the total protein is < 2.5 g/dL (<25 g/L), whereas in cardiac ascites, it is ≥ 2.5 g/dL (≥25 g/L). In addition, N-terminal pro-brain-type natriuretic peptide (NT-proBNP) in serum can be used as a marker to help distinguish ascitic fluid due to heart failure from that due to cirrhosis. In a retrospective study, median values of NT-proBNP were significantly higher in patients with heart failure compared with cirrhosis (6100 vs 166 pg/mL, respectively). Patients with both heart failure and cirrhosis have values in the heart failure range.^[13]^ Ascitic fluid in our patient was characterized by high SAAG and high ascitic fluid total protein, along with high serum NT-proBNP level.

Although cardiac ascites has a higher RBC count in ascitic fluid compared with cirrhotic ascites,^[10,14]^ grossly bloody ascites secondary to HF is extremely rare. To our knowledge, only one case describing hemorrhagic ascites secondary to HF was reported in the literature to date.^[8]^ Hemorrhagic ascites is described as ascitic fluid with RBC count of at least 10,000/mm^3^,^[6,7]^ while 50,000/mm^3^ is the cutoff in some references.^[15]^ RBC count in the ascitic fluid of our patient was persistently > 75,000/mm^3^ in 2 samples over 2 months. The underlying mechanism of bloody ascites related to HF is unsettled, but it is reasonable to expect a mechanism similar to that of spontaneous hemorrhagic ascites related to cirrhosis. It is likely to be due to one of two processes: (1) leakage from the interstitial spaces of the liver or splanchnic bed in a manner similar to red cell diapedesis in systemic venous hypertension or (2) intra-abdominal bleeding from a small vessel (likely thin-walled, portosystemic collateral) or an abdominal cavity varix.^[7,16]^

The presence of hemorrhagic ascites in patients with cirrhosis has been shown to be a poor prognostic marker and is associated with increased mortality.^[17]^ Although cirrhosis accounts for ≈80% of all ascites cases,^[2]^ only 5% to 19% of these cases are hemorrhagic.^[7,18]^ Other causes of hemorrhagic ascites frequently encountered in practice include infections (e.g. abdominal tuberculosis) and iatrogenic following trauma to the liver or spleen (eg, after liver biopsy or laparoscopy). In addition, malignancy (from different tumor origins) is a well-known cause of hemorrhagic ascites. In a prospective study of 45 patients with malignancy-related ascites, 20% of ascitic fluid samples were hemorrhagic.^[19]^ Infrequent causes reported in the literature include sarcoidosis,^[20]^ endometriosis,^[21]^ and ruptured ovarian cyst.^[22]^

It is essential to rule out other common and serious causes before attributing the cause of hemorrhagic ascites to HF. In our patient, peritoneal fluid examinations for AFB, malignant cytology, and bacterial cultures were negative in 3 different samples. Serum inflammatory markers were normal, as were tumor markers, such as AFP, carcinoembryonic antigen, and cancer antigen 19-9. A whole-body computed tomography scan with contrast was not done due to high creatinine and risk of contrast-induced nephropathy. Instead, a PET scan was done to rule out secondary causes of hemorrhagic ascites, particularly malignancy and infection, and it was unremarkable.

Management of hemorrhagic ascites depends on the underlying cause. Due to its rarity, there are no clear guidelines for managing hemorrhagic ascites related to HF. Goel et al^[8]^ described a case of hemorrhagic ascites managed successfully with paracentesis and decongestive therapy with a clearance of hemorrhagic ascites on repeated paracentesis. Our patient was managed similarly in the first presentation; however, hemorrhagic ascites persisted on repeated paracentesis. In the second presentation, she was managed again with therapeutic ascitic drainage, aggressive diuresis, and optimization of her HF medications. The patient reported improvement of her symptoms and was discharged in a stable condition, yet her ascitic fluid continued to be frankly hemorrhagic until the end and did not clear.

## 4. Conclusions

Hemorrhagic ascites is often an alarming finding to most physicians due to potentially serious causes, especially malignancy. However, in patients having features of heart failure, high hydrostatic pressure in the setting of right-sided heart failure should be kept in the differential diagnosis of hemorrhagic ascites after ruling out other causes.

## Acknowledgments

The authors would like to acknowledge the Qatar National Library for funding the open access fees of this publication.

## Author contributions

SA: conceptualization, writing - original draft. AF and NE: participated in literature review and reviewing the manuscript; MBH: writing - review and editing.
